# Risk factors, prognostic potency, and longitudinal variation of anxiety and depression in postoperative glioma patients

**DOI:** 10.3389/fsurg.2022.1069709

**Published:** 2023-01-16

**Authors:** Xiaohua Wu, Dongdong Wang, Dan Wang

**Affiliations:** ^1^ Department of Neurosurgery IV, The Second Affiliated Hospital of Harbin Medical University, Harbin, China; ^2^ Department of Cardiology, The Second Affiliated Hospital of Harbin Medical University, Harbin, China; ^3^ Department of Neurosurgery II, The Second Affiliated Hospital of Harbin Medical University, Harbin, China

**Keywords:** anxiety and depression, glioma, risk factors, overall survival, longitudinal variation

## Abstract

**Objective:**

Anxiety and depression are common mental disorders in glioma patients. This study aimed to evaluate the risk factors, prognostic role, and longitudinal changes in anxiety and depression in postoperative glioma patients.

**Methods:**

Anxiety and depression were assessed by Hospital Anxiety and Depression Scale at baseline, month (M) 6, M12, M24 and M36 in 270 glioma patients after surgical resection. Furthermore, comprehensive clinic characteristics and treatment-related information were collected.

**Results:**

Gender (female vs. male) (*P *= 0.014, odds ratio (OR) = 1.974), marital status (single/divorced/widowed vs. married) (*P *= 0.019, OR = 2.172), Karnofsky performance status (KPS) score (≤70 vs. > 70) (*P *= 0.002, OR = 2.556), World Health Organization (WHO) classification (high-grade glioma (HGG) vs. low-grade glioma (LGG)) (*P *= 0.005, OR = 2.155), and postoperative complications (yes vs. not) (*P *= 0.001, OR = 2.525) were independently related to anxiety occurrence. Marital status (single/divorced/widowed vs. married) (*P *= 0.034, OR = 2.026), KPS score (≤70 vs. > 70) (*P *< 0.001, OR = 3.880), WHO classification (HGG vs. LGG) (*P *= 0.032, OR = 1.810), and postoperative complications (yes vs. not) (*P *= 0.001, OR = 2.602) were independently related to depression occurrence. Besides, anxiety (*P *= 0.038) and depression (*P *= 0.013) were linked with shorter overall survival (OS), and depression was an independent risk factor for worse OS (*P *= 0.040, hazard ratio = 1.596). More importantly, anxiety and depression remained at a high prevalence during a 3-year follow-up.

**Conclusion:**

Gender, marital status, KPS score, WHO classification, and postoperative complications are risk factors for anxiety and depression; moreover, anxiety and depression are at high prevalence continuously and correlated with worse survival in postoperative glioma patients.

## Introduction

Glioma is the most common primary central nervous system tumor, with high aggressiveness and excessive mortality (1, 2). At present, the main treatment for glioma is surgical resection, and other treatments include radiation therapy, chemotherapy, and emerging immunotherapy (3–5). Even with these treatments, the prognosis of glioma patients is still unfavorable (6, 7). According to previous studies, the median survival time for patients with low-grade glioma (LGG) (World Health Organization (WHO) I-II grade) is 5.6–13.3 years, while for patients with high-grade glioma (HGG) (WHO III-IV grade), it is only 12.2–15.4 months (7–10). Poor survival of glioma patients may lead to a heavy mental burden (11, 12). Apart from that, glioma patients face some other problems: for example, areas of brain impairment could cause a high risk of mental disorders (13, 14). Moreover, treatments of glioma (such as surgery, chemotherapy, etc.) may impose negative influences on their mental health, too (15, 16). Therefore, the mental health of glioma patients is a major issue currently.

In order to better manage the mental health of glioma patients, many studies have recognized potential factors that are associated with anxiety and depression in these patients (11, 12, 17). One previous study indicates blood inflammatory cytokines could be predictors of depression in glioma patients (17). Moreover, another study suggests that decreased IL-2 levels and elevated IFN-*γ* levels were positively associated with anxiety and depression in glioma patients, respectively (11). Significantly, a recent study has indicated that female gender, single, divorced, or widowed marital status, increased WHO classification, shorter education duration, chronic kidney disease (CKD), and hyperlipidemia are risk factors for anxiety and depression in glioma patients (12). However, this study has a small sample size (*N* = 190) and does not evaluate the longitudinal variation of anxiety and depression in glioma patients; at the same time, some important factors affecting anxiety and depression are not included in its study (such as Karnofsky performance status (KPS) score, postoperative complications, etc.) and the prognostic role of anxiety and depression is not adjusted by multivariate regression analysis in glioma patients. Our study hypothesized that anxiety and depression were at high prevalence in postoperative glioma patients and had important impacts on their prognosis. In addition, there might be some risk factors obviously related to anxiety and depression in postoperative glioma patients, such as gender and postoperative complications, etc. These risk factors might be beneficial to the management of postoperative glioma patients. In addition, the Glioma Outcomes (GO) Project provides high-standard care for postoperative glioma patients (18).

Therefore, the present study included 270 glioma patients who received surgical resection and collected sufficient characteristics and treatment information, aiming to comprehensively evaluate the risk factors and prognostic value of anxiety and depression, as well as to assess the change in anxiety and depression longitudinally in those patients.

## Methods

### Subjects

From March 2017 to November 2021, 270 glioma patients who received surgical resection were consecutively recruited. The inclusion criteria were: (1) diagnosed as glioma; (2) had surgical resection; (3) able to complete assessment of Hospital Anxiety and Depression Scale (HADS); (4) had more than 3 months of life expectancy; (5) older than 18 years. The exclusion criteria were: (1) cognitive impairments; (2) history of other primary malignancies; (3) pregnant or lactating women. The Ethics Committee of The Second Affiliated Hospital of Harbin Medical University permitted the study ethic. All participants signed the written informed consent.

### Data collection

After enrollment, the patients' demographics, underlying diseases, features of glioma, and treatment-related information were collected from the case report form. The demographics included age, gender, education level, marital status, preoperative employment status, and location. The underlying diseases included hypertension, hyperlipidemia, and diabetes. The features of glioma included KPS score, WHO classification, isocitrate dehydrogenase (IDH) mutation, and tumor location. The treatment-related information included postoperative complications, adjuvant radiotherapy, and adjuvant chemotherapy. The postoperative complications were assessed based on a previous study, which included direct cortical and vascular injury, surgical wound complications, and postsurgical medical complications (19).

### HADS assessment

A total of 270 glioma patients completed the HADS questionnaire at baseline (discharge after surgery). Furthermore, patients had evaluation of HADS at 6 months (M6), 12 months (M12), 24 months (M24), and 36 months (M36) after discharge. For the different patients' follow-up duration, the number of patients who had HADS evaluation varied at different time points. The definition of HADS was consistent with the previous study (20). Anxiety or depression was defined as having a HADS-anxiety (HADS-A) or HADS-depression (HADS-D) score more than 7 (20).

### Follow-up

After discharge, all patients were followed up until death or lost to follow-up. The last follow-up date was April 2022. The overall survival (OS) was computed from the day of resection to the day of death. The range of follow-up was 2.3 to 49.9 months.

### Statistical analysis

The statistical analyses were conducted by SPSS v27.1 (IBM Corp., United States). The figures were plotted *via* GraphPad Prism v8.01 (GraphPad Software Inc., United States). Risk factors of anxiety or depression were assessed *via* univariable and multivariate logistic regression model with step forward methods, and all factors shown in the univariable logistic regression model were included in the multivariate logistic regression model. The difference in survival data between groups was displayed using Kaplan-Meier curves and analyzed log-rank test. Factors related to OS were evaluated through univariable and multivariate Cox's regression model with step forward methods, and all factors shown in the univariable Cox's regression model were included in the multivariate Cox's regression model. The change of HADS score was tested by analysis of variance (ANOVA) for repeated measurements. The change in anxiety or depression rate was tested by the Chi-square test for trend. *P* < 0.05 was considered significant.

## Results

### Study flow

**Figure 1:**
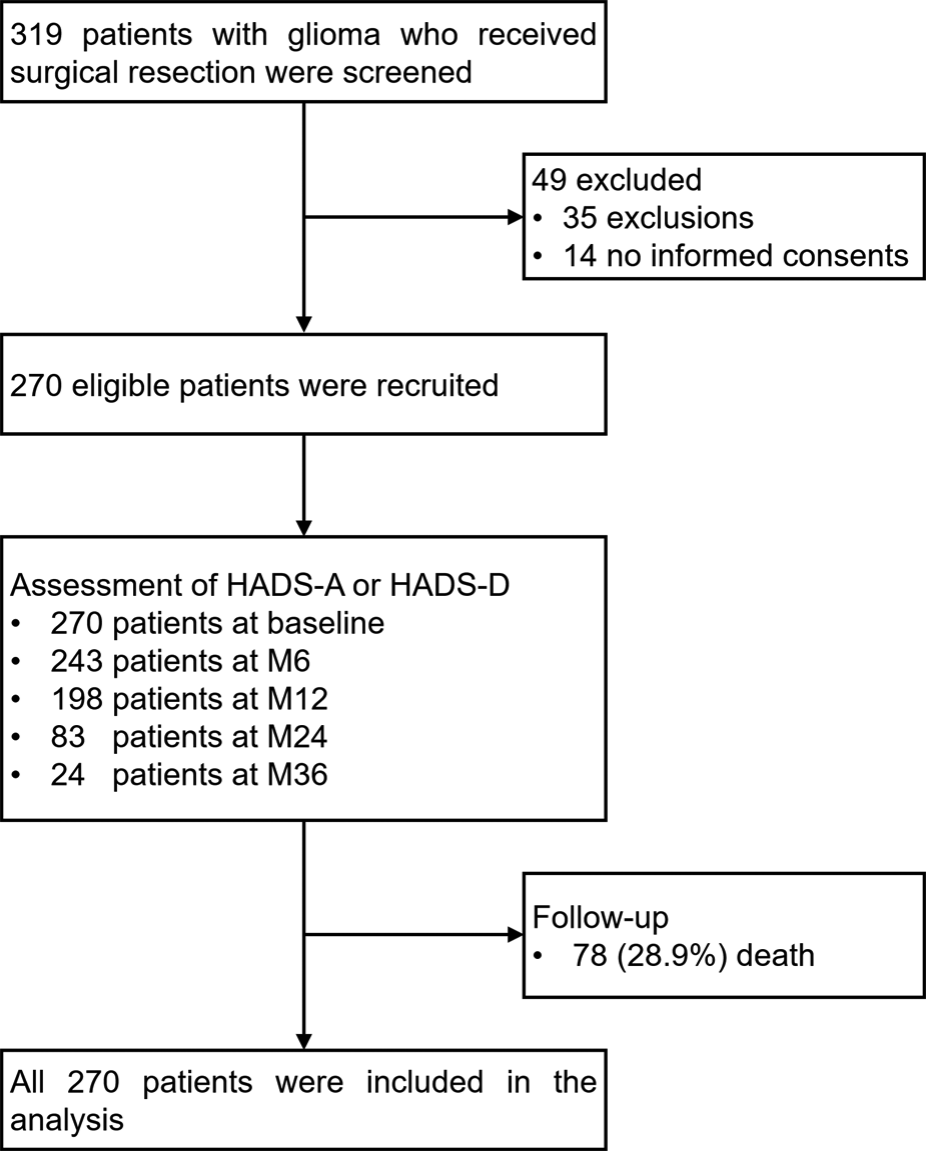
Study flow chart.

Totally, 319 glioma patients who received surgical resection were screened, among which 49 patients were excluded, consisting of 35 patients who either met the exclusion criteria or did not meet the inclusion criteria and 14 patients who refused to sign informed consents. The rest 270 eligible patients were recruited, and then 270, 243, 198, 83 and 24 patients completed the assessment of HADS-A or HADS-D at baseline, M6, M12, M24, and M36, respectively. Meanwhile, 78 (28.9%) patients died during the follow-up period. Finally, all 270 patients were included in the analysis based on intention-to-treat (ITT) approaches (Figure 1).

### Baseline characteristics of glioma patients

The glioma patients included 94 (34.8%) females and 176 (65.2%) males with a mean age of 48.0 ± 10.8 years. There were 129 (47.8%) patients with IDH mutation and 141 (52.2%) patients without that. Besides, the mean value of KPS score in glioma patients was 71.0 ± 9.7. As to WHO classification, there were 122 (45.2%) patients classified as LGG and 148 (54.8%) patients classified as HGG. Meanwhile, all glioma patients underwent surgical resection, and 82 (30.4%) patients had postoperative complications. In addition, the mean values of HADS-A and HADS-D scores at baseline in glioma patients were 8.0 ± 3.4 and 7.7 ± 3.2, respectively. More detailed characteristics of glioma patients were described in Table 1.

**Table 1:** Baseline characteristics.

Items	Patients with glioma (*N* = 270)
Age (years), mean ± SD	48.0 ± 10.8
Gender, *n* (%)	
Female	94 (34.8)
Male	176 (65.2)
Education level, *n* (%)	
Primary school or below	26 (9.6)
Middle or high school	189 (70.0)
Undergraduate or above	55 (20.4)
Marital status, *n* (%)	
Married	218 (80.7)
Single/divorced/widowed	52 (19.3)
Preoperative employment status, *n* (%)	
Employed	204 (75.6)
Unemployed	66 (24.4)
Location, *n* (%)	
Urban	218 (80.7)
Rural	52 (19.3)
Hypertension, *n* (%)	73 (27.0)
Hyperlipidemia, *n* (%)	37 (13.7)
Diabetes, *n* (%)	30 (11.1)
KPS score, mean ± SD	71.0 ± 9.7
WHO classification, *n* (%)	
LGG	122 (45.2)
HGG	148 (54.8)
IDH mutation, *n* (%)	
No	141 (52.2)
Yes	129 (47.8)
Tumor location, *n* (%)	
Frontal	112 (41.5)
Temporal	63 (23.3)
Parietal	45 (16.7)
Occipital	18 (6.7)
Posterior fossae	10 (3.7)
Others	22 (8.1)
Surgical resection, *n* (%)	270 (100.0)
Postoperative complications, *n* (%)	82 (30.4)
Adjuvant radiotherapy, *n* (%)	164 (60.7)
Adjuvant chemotherapy, *n* (%)	112 (41.5)
HADS-A score, mean ± SD	8.0 ± 3.4
Anxiety, *n* (%)	
No	160 (59.3)
Yes	110 (40.7)
HADS-D score, mean ± SD	7.7 ± 3.2
Depression, *n* (%)	
No	170 (63.0)
Yes	100 (37.0)

SD, standard deviation; KPS, Karnofsky performance status; WHO, World Health Organization; LGG, low grade glioma; HGG, high grade glioma; IDH, isocitrate dehydrogenase; HADS, Hospital Anxiety and Depression Scale.

### Factors correlated with anxiety in glioma patients

Univariate logistic regression model exhibited that gender (female vs. male) (*P *= 0.024, odds ratio (OR) = 1.794), marital status (single/divorced/widowed vs. married) (*P *= 0.015, OR=2.133), KPS score (≤70 vs. > 70) (*P *= 0.015, OR = 1.945), WHO classification (HGG vs. LGG) (*P *= 0.016, OR=1.840), tumor location (parietal vs. frontal) (*P *= 0.001, OR=3.207) and postoperative complications (yes vs. not) (*P *= 0.005, OR=2.140) were associated with higher risk of anxiety at baseline in glioma patients. Next, analysis by multivariate logistic regression model revealed that gender (female vs. male) (*P *= 0.010, OR=2.100), marital status (single/divorced/widowed vs. married) (*P *= 0.017, OR=2.262), KPS score (≤70 vs. > 70) (*P *= 0.003, OR=2.535), WHO classification (HGG vs. LGG) (*P *= 0.005, OR=2.239), tumor location (parietal vs. frontal) (*P *= 0.003, OR=3.267), and postoperative complications (yes vs. not) (*P *= 0.003, OR=2.406) were independent risk factors for anxiety at baseline in glioma patients (Table 2).

**Table 2:** Risk factors of anxiety by logistic regression model analysis.

Items	*P* value	OR	95% CI	
			Lower	Upper
**Univariate logistic regression**				
Age (≥50 years vs. < 50 years)	0.087	1.532	0.939	2.497
Gender (Female vs. Male)	0.024	1.794	1.079	2.982
Higher education level	0.182	0.732	0.463	1.157
Marital status (Single/divorced/widowed vs. Married)	0.015	2.133	1.156	3.934
Preoperative employment status (Unemployed vs. Employed)	0.237	1.401	0.801	2.452
Location (Rural vs. Urban)	0.377	1.316	0.715	2.419
Hypertension (Yes vs. No)	0.364	1.286	0.747	2.212
Hyperlipidemia (Yes vs. No)	0.160	1.647	0.821	3.305
Diabetes (Yes vs. No)	0.140	1.775	0.828	3.806
KPS score (≤70 vs. > 70)	0.015	1.945	1.135	3.332
WHO classification (HGG vs. LGG)	0.016	1.840	1.119	3.024
IDH mutation (Yes vs. No)	0.169	0.710	0.435	1.157
Tumor location				
Frontal	Ref.			
Temporal	0.447	1.281	0.677	2.426
Parietal	0.001	3.207	1.564	6.579
Occipital	0.389	1.558	0.568	4.271
Posterior fossae	0.315	1.947	0.531	7.144
Others	0.544	0.730	0.264	2.018
Postoperative complications (Yes vs. Not)	0.005	2.140	1.263	3.627
Adjuvant radiotherapy (Yes vs. No)	0.189	1.400	0.847	2.315
Adjuvant chemotherapy (Yes vs. No)	0.110	1.494	0.913	2.445
**Multivariate logistic regression**				
Gender (Female vs. Male)	0.010	2.100	1.190	3.704
Marital status (Single/divorced/widowed vs. Married)	0.017	2.262	1.154	4.433
KPS score (≤70 vs. > 70)	0.003	2.535	1.382	4.650
WHO classification (HGG vs. LGG)	0.005	2.239	1.283	3.909
Tumor location				
Frontal	Ref.			
Temporal	0.384	1.361	0.680	2.722
Parietal	0.003	3.267	1.515	7.041
Occipital	0.588	1.351	0.455	4.008
Posterior fossae	0.564	1.511	0.372	6.134
Others	0.509	0.697	0.238	2.037
Postoperative complications (Yes vs. Not)	0.003	2.406	1.351	4.284

OR, odds ratio; CI, confidence interval; KPS, Karnofsky performance status; WHO, World Health Organization; LGG, low grade glioma; HGG, high grade glioma; IDH, isocitrate dehydrogenase.

### Factors correlated with depression in glioma patients

By univariate logistic regression model, it was observed that marital status (single/divorced/widowed vs. married) (*P *= 0.033, OR=1.946), KPS score (≤70 vs. > 70) (*P *< 0.001, OR=2.984), tumor location (parietal vs. frontal) (*P *= 0.018, OR=2.340) and postoperative complications (yes vs. not) (*P *= 0.004, OR=2.186) were related to increased risk of depression at baseline in glioma patients. Moreover, marital status (single/divorced/widowed vs. married) (*P *= 0.034, OR=2.026), KPS score (≤70 vs. > 70) (*P *< 0.001, OR=3.880), WHO classification (HGG vs. LGG) (*P *= 0.032, OR=1.810), and postoperative complications (yes vs. not) (*P *= 0.001, OR=2.602) were independent risk factors for depression at baseline in glioma patients (Table 3).

**Table 3:** Risk factors of depression by logistic regression model analysis.

Items	*P* value	OR	95% CI	
			Lower	Upper
**Univariate logistic regression**				
Age (≥50 years vs. < 50 years)	0.104	1.511	0.919	2.483
Gender (Female vs. Male)	0.171	1.432	0.856	2.395
Higher education level	0.381	0.813	0.512	1.291
Marital status (Single/divorced/widowed vs. Married)	0.033	1.946	1.056	3.587
Preoperative employment status (Unemployed vs. Employed)	0.298	1.351	0.767	2.380
Location (Rural vs. Urban)	0.382	1.316	0.711	2.437
Hypertension (Yes vs. No)	0.578	1.170	0.674	2.031
Hyperlipidemia (Yes vs. No)	0.118	1.745	0.868	3.508
Diabetes (Yes vs. No)	0.249	1.567	0.730	3.365
KPS score (≤70 vs. > 70)	<0.001	2.984	1.662	5.360
WHO classification (HGG vs. LGG)	0.118	1.492	0.903	2.465
IDH mutation (Yes vs. No)	0.484	0.838	0.510	1.375
Tumor location				
Frontal	Ref.			
Temporal	0.989	1.004	0.526	1.919
Parietal	0.018	2.340	1.156	4.735
Occipital	0.559	0.720	0.239	2.168
Posterior fossae	0.743	1.248	0.332	4.688
Others	0.274	0.551	0.189	1.605
Postoperative complications (Yes vs. Not)	0.004	2.186	1.285	3.719
Adjuvant radiotherapy (Yes vs. No)	0.401	1.244	0.747	2.072
Adjuvant chemotherapy (Yes vs. No)	0.248	1.342	0.814	2.213
**Multivariate logistic regression**				
Marital status (Single/divorced/widowed vs. Married)	0.034	2.026	1.056	3.886
KPS score (≤70 vs. > 70)	<0.001	3.880	2.067	7.283
WHO classification (HGG vs. LGG)	0.032	1.810	1.053	3.113
Postoperative complications (Yes vs. Not)	0.001	2.602	1.469	4.610

OR, odds ratio; CI, confidence interval; KPS, Karnofsky performance status; WHO, world health organization; LGG, low grade glioma; HGG, high grade glioma; IDH, isocitrate dehydrogenase.

### Association of anxiety and depression with OS in glioma patients

**Figure 2:**
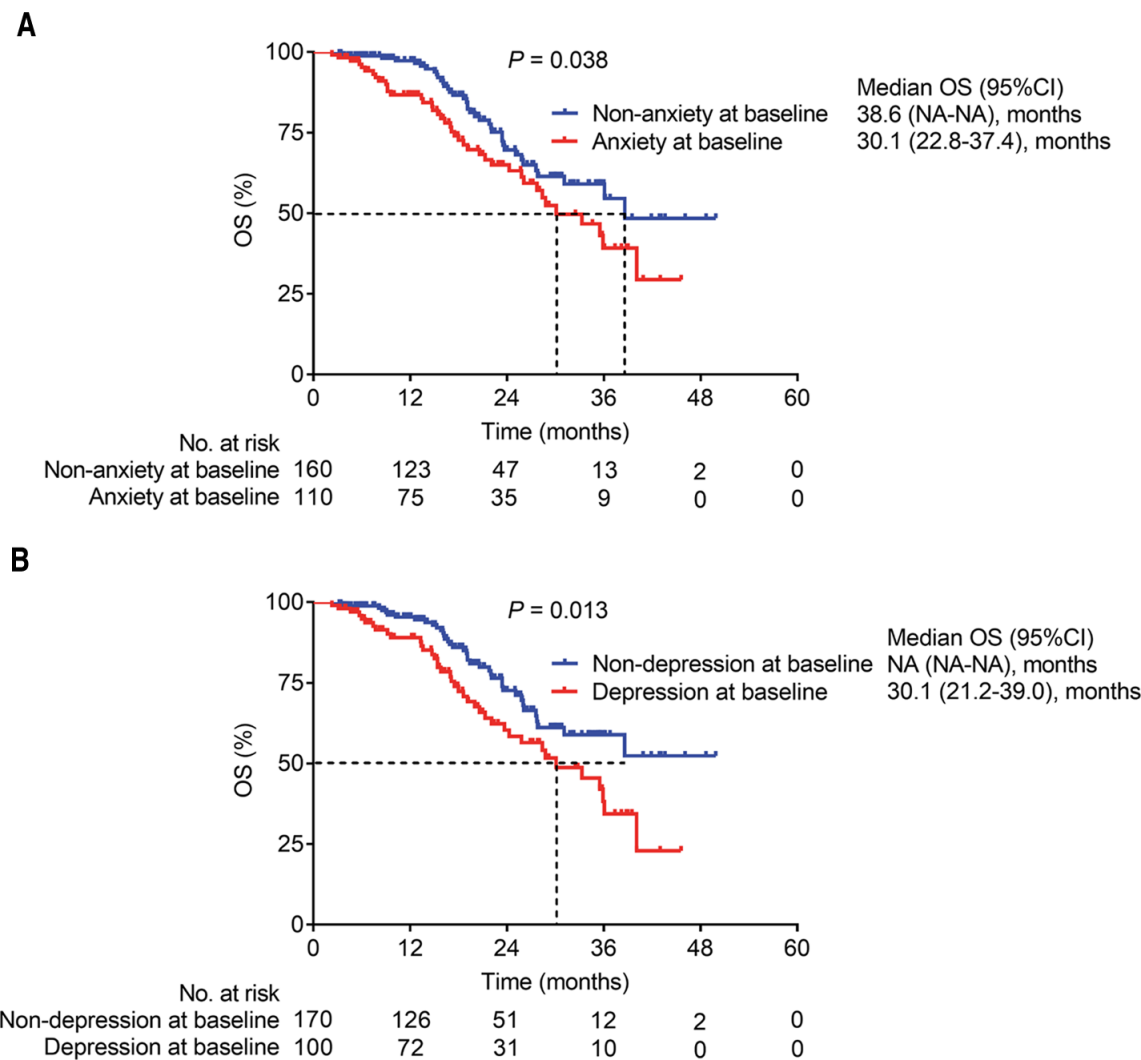
The relationship of anxiety and depression with OS in glioma patients. OS in glioma patients with anxiety and those without anxiety at baseline (**A**); and OS in glioma patients with depression and those without depression at baseline (**B**).

The OS was reduced in patients with anxiety at baseline (median (95% confidence interval (CI)): 30.1 (22.8–37.4) months) compared to patients without anxiety at baseline (median (95% CI): 38.6 (NA-NA) months) (*P *= 0.038) (Figure 2A). In terms of depression, OS was decreased in patients with depression at baseline (median (95% CI): 30.1 (21.2–39.0) months) compared with those without depression at baseline (median (95% CI): NA (NA-NA) months) (*P *= 0.013) (Figure 2B).

### Factors correlated with OS in glioma patients

**Figure 3:**
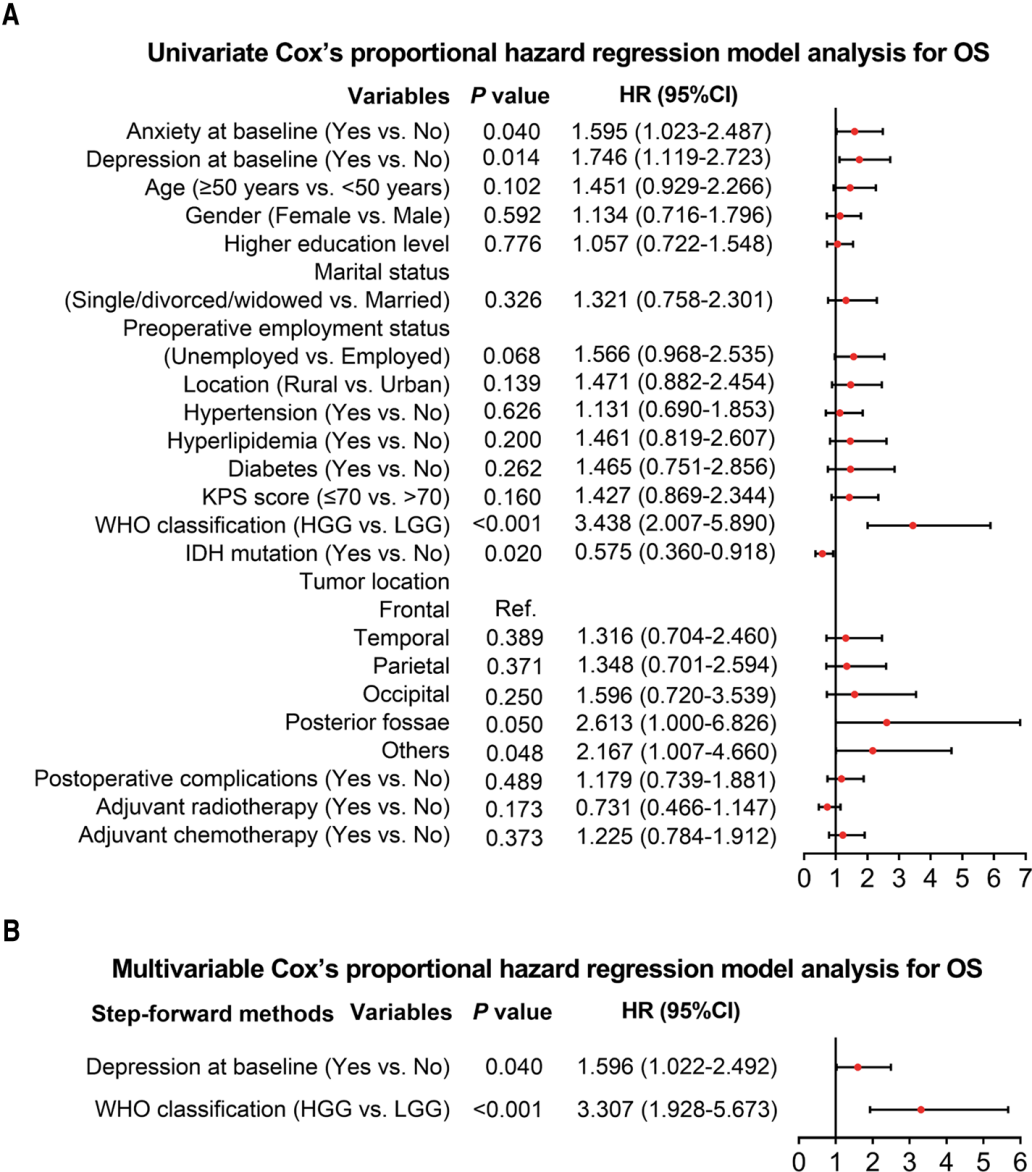
Cox's proportional hazard regression for factors influencing OS in glioma patients. Factors related to OS in glioma patients by univariate Cox's proportional hazard regression (**A**). Independent factors that affect OS in glioma patients by multivariate Cox's proportional hazard regression (**B**).

Univariate Cox's proportional hazard regression model was subsequently used to evaluate factors that influenced OS, which indicated that anxiety at baseline (yes vs. no) (*P *= 0.040, hazard ratio (HR) = 1.595), depression at baseline (yes vs. no) (*P *= 0.014, HR = 1.746), WHO classification (HGG vs. LGG) (*P *< 0.001, HR = 3.438), and tumor location (others vs. frontal) (*P *= 0.048, HR = 2.167) were linked with worse OS; while IDH mutation (yes vs. no) was correlated with better OS (*P *= 0.020, HR = 0.575) in glioma patients (Figure 3A). Further multivariate Cox's proportional hazards regression model showed that depression at baseline (yes vs. no) (*P *= 0.040, HR = 1.596) and WHO classification (HGG vs. LGG) (*P *< 0.001, HR = 3.307) were independent risk factors for worse OS in glioma patients (Figure 3B).

### The longitudinal change in anxiety and depression in glioma patients

**Figure 4:**
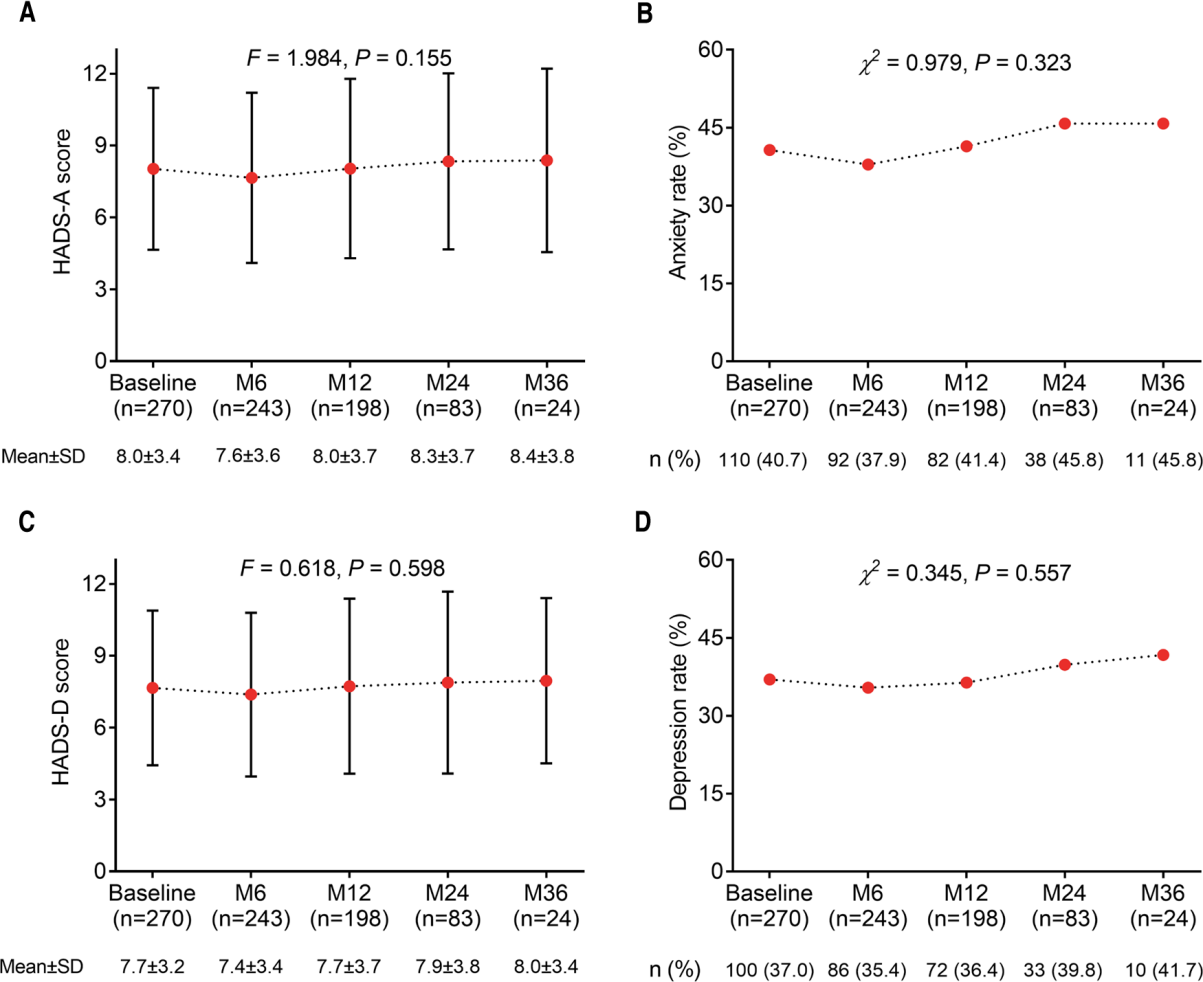
Changes in anxiety and depression with different time points in postoperative glioma patients. The HADS-A score (**A**), anxiety rate (**B**), HADS-D score (**C**), and depression rate (**D**) among each time point in glioma patients.

There was no difference in HADS-A score (*P *= 0.155) (Figure 4A) and anxiety rate (*P *= 0.323) (Figure 4B) among any time points in glioma patients. Similarly, no difference in HADS-D score (*P *= 0.598) (Figure 4C) and depression rate (*P *= 0.557) (Figure 4D) was found among any time points in glioma patients. In addition, the last observation carried forward (LOCF) analysis showed that there was no difference in HADS-A score (*P *= 0.091) (Supplementary Figure S1A) and anxiety rate (*P *= 0.594) (Supplementary Figure S1B) at different time points in glioma patients; moreover, HADS-D score (*P *= 0.024) (Supplementary Figure S1C) increased continually while there was no distinction of depression rate (*P *= 0.931) (Supplementary Figure S1D) at different time points in glioma patients.

## Discussion

Anxiety and depression are common clinical symptoms in glioma patients who received surgical resection, which should be paid high attention since they have many negative influences, for example, reducing the quality of life, worsening the performance status of glioma patients, etc (21, 22).. However, only a small number of studies have reported the risk factors of anxiety and depression in glioma patients. For instance, one study finds that elevated IFN-*γ* levels are positively related to depression in glioma patients (11). Another literature states that anxiety is more common in younger glioma patients than in older glioma patients (23). Additionally, a recent article illustrates several factors for anxiety and depression in glioma patients, including gender, marital status, WHO classification, hyperlipidemia, education duration, and CKD (12). However, this previous study has some shortages as described in this introduction section. In our study, it was revealed that gender, marital status, KPS score, WHO classification, tumor location, and postoperative complications were independent risk factors for anxiety in glioma patients. Furthermore, marital status, KPS score, WHO classification, and postoperative complications were independent risk factors for depression in glioma patients. These findings could be explained by that: (a) Estrogen imbalance could induce the females-specific risk of mental disorders (24), so females might be more prone to anxiety than males in glioma patients. However, this is a conjecture and needs to be verified in further study. (b) Single/divorced/widowed glioma patients might feel lonelier and more emotionally compared with those with married status (25, 26). Therefore, glioma patients with single/divorced/widowed marital status might be at a high risk of anxiety and depression. (c) Glioma patients with KPS score ≤ 70 had poor performance status and were unable to take care of themselves, both events could cause anxiety and depression. (d) The recurrence risk is higher in HGG patients than in LGG patients (27), and thus they might feel more fear of recurrence than LGG patients (which was positively correlated with anxiety and depression (28)), so WHO classification was a risk factor for anxiety and depression in glioma patients. (e) The parietal was an important region for anxious arousal, whose impairment of function might cause mental disorders in glioma patients (29, 30), and thus parietal tumor was a risk factor for anxiety in glioma patients. (f) The postoperative complications (including epilepsy and cognitive impairment, etc.) might directly cause anxiety and depression in glioma patients (31–33). Therefore, postoperative complications appear to be risk factors for anxiety and depression in glioma patients.

Anxiety and depression could have serious negative consequences on the prognosis of cancer patients. Evidence shows that anxiety and depression are positively associated with mortality in patients with cancers, including glioma (12, 34, 35).. For example, one study suggests that glioma patients with depression have worse OS than those without depression (36). Another research indicates a worse OS in glioma patients with depression, especially in HGG glioma patients (35). In addition, a recent study has also shown that anxiety and depression are related to worse survival in glioma patients (12). The findings of these previous studies are in part consistent with the results of our study, which revealed that anxiety or depression at baseline was associated with poorer OS in glioma patients. The explanations for these results are as follows: (a) Depression might affect the endocrine system of the patients, thereby indirectly accelerating the deterioration of the condition, which caused a worse OS (37). (b) Anxiety and depression might make patients reluctant to cooperate with post-operative recovery treatment and even lead to suicide (38).

Clearly, the longitudinal progress of mental disorders in cancer patients is also a notable issue. For instance, a previous study reports the prevalence of depression has been continuously high during a 1-year period in cancer patients (39). It is partly consistent with our results. Our study performed longitudinal assessments and found that anxiety and depression remained high during a 3-year period in glioma patients, while they did not rise significantly. This might be because: Glioma patients with anxiety and depression were at a high risk of death (12, 35, 36), implying that patients with high anxiety and depression died possibly at the follow-up period over time. In our study, the deaths of these patients might lead to the underestimation of anxiety and depression during the follow-up period in glioma patients.

In addition, several study designs should be pointed out as well in the current study: (1) Our study chose HADS because of its simplicity, convenience, and heterogeneity. Our study needed to evaluate anxiety and depression in glioma patients at multiple time points, so it was easier to implement the study design by using simple scales to evaluate anxiety and depression in glioma patients, such as HADS. (2) In fact, some factors might be potentially related. For example, we thought that the WHO grade and A/D were likely to interfere with each other. However, their interference was not direct. Therefore, if the WHO grade had been excluded, the results of our study might be misjudged. In order to reflect the prognosis role of anxiety and depression in postoperative glioma patients in an objective manner as much as possible, we considered including WHO grade as a variable.

This study, however, still presents some limitations: (a) It is a single-center study, which leads to selection bias. (b) It only includes adult patients with glioma, however, the situation of children with glioma is unclear, and further research should be conducted to evaluate risk factors and prognostic potency of anxiety and depression in children with glioma. (c) It only assesses anxiety and depression in glioma patients by HADS, and future studies should use multiple assessment scales to further investigate their anxiety and depression.

In conclusion, our study discovers multiple independent risk factors for anxiety and depression in glioma patients who receive surgical resection. Moreover, anxiety and depression have a high prevalence and are connected with poorer survival in those patients. These findings may improve the management of mental health and prognosis of glioma patients.

## Data Availability

The original contributions presented in the study are included in the article/**Supplementary Material**, further inquiries can be directed to the corresponding author/s.
