# Supplementary material for: Risk factors, prognostic potency, and longitudinal variation of anxiety and depression in postoperative glioma patients
Source: Front Surg. 2023 Jan 16;9:1069709. doi: 10.3389/fsurg.2022.1069709 (PMC9884976; doi:10.3389/fsurg.2022.1069709)
Supplement: Supplementary file 1 [file Datasheet1.pdf]

Supplementary Materials

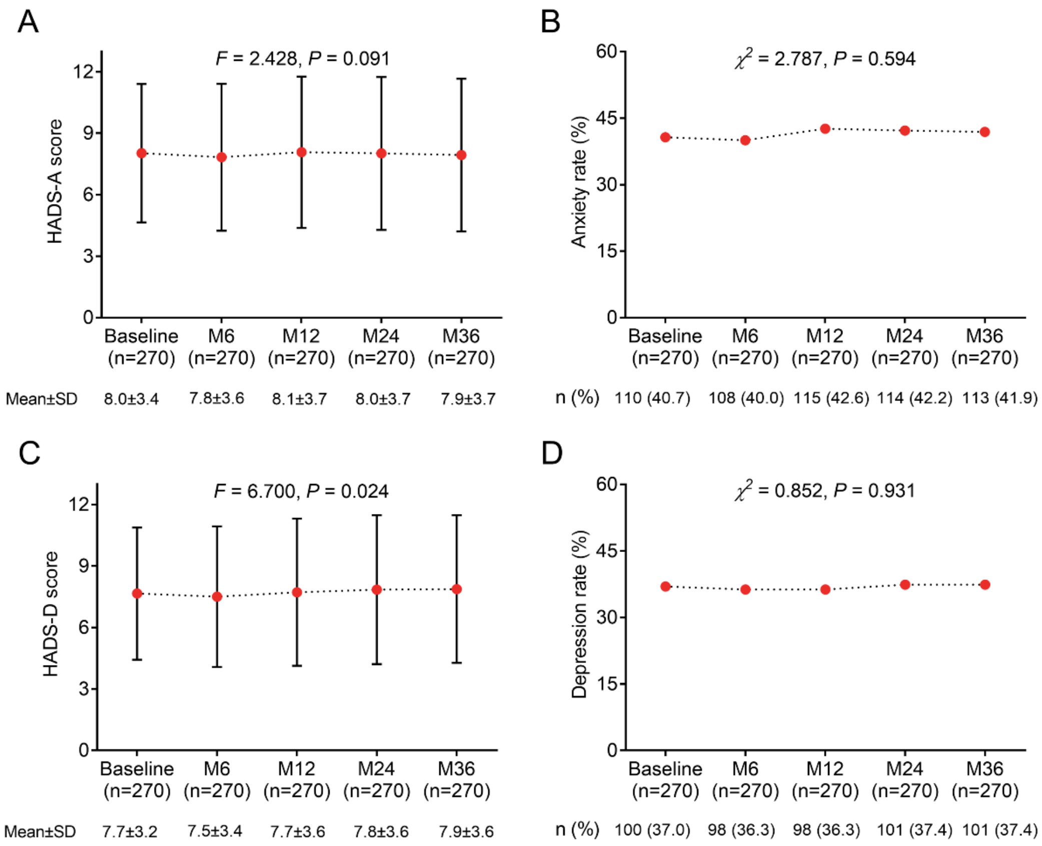

Supplementary Figure 1. Changes in anxiety and depression with different time points in postoperative glioma patients by LOCF analysis. The HADS-A score (A), anxiety rate (B), HADS-D score (C), and depression rate (D) among each time point in glioma patients by LOCF analysis.
